# Single-Walled Carbon Nanotubes Inhibit the Cytochrome P450 Enzyme, CYP3A4

**DOI:** 10.1038/srep21316

**Published:** 2016-02-22

**Authors:** Ramy El-Sayed, Kunal Bhattacharya, Zonglin Gu, Zaixing Yang, Jeffrey K. Weber, Hu Li, Klaus Leifer, Yichen Zhao, Muhammet S. Toprak, Ruhong Zhou, Bengt Fadeel

**Affiliations:** 1 Nanosafety & Nanomedicine Laboratory, Division of Molecular Toxicology, Institute of Environmental Medicine, Karolinska Institutet, 17177 Stockholm, Sweden; 2 Institute of Quantitative Biology and Medicine, Collaborative Innovation Center of Radiation Medicine of Jiangsu Higher Education Institutions, Soochow University, Suzhou 215123, China; 3 IBM Thomas J. Watson Research Center, Yorktown Heights, New York 10598, USA; 4 Department of Engineering Sciences, Uppsala University, 75121 Uppsala, Sweden; 5 Functional Materials Division, Department of Materials and Nanophysics, KTH-Royal Institute of Technology, 16440 Stockholm, Sweden; 6 Department of Chemistry, Columbia University, New York, New York 10027, USA; 7 Department of Environmental and Occupational Health, University of Pittsburgh, Pittsburgh, Pennsylvania 15219, USA

## Abstract

We report a detailed computational and experimental study of the interaction of single-walled carbon nanotubes (SWCNTs) with the drug-metabolizing cytochrome P450 enzyme, CYP3A4. Dose-dependent inhibition of CYP3A4-mediated conversion of the model compound, testosterone, to its major metabolite, 6β-hydroxy testosterone was noted. Evidence for a direct interaction between SWCNTs and CYP3A4 was also provided. The inhibition of enzyme activity was alleviated when SWCNTs were pre-coated with bovine serum albumin. Furthermore, covalent functionalization of SWCNTs with polyethylene glycol (PEG) chains mitigated the inhibition of CYP3A4 enzymatic activity. Molecular dynamics simulations suggested that inhibition of the catalytic activity of CYP3A4 is mainly due to blocking of the exit channel for substrates/products through a complex binding mechanism. This work suggests that SWCNTs could interfere with metabolism of drugs and other xenobiotics and provides a molecular mechanism for this toxicity. Our study also suggests means to reduce this toxicity, *eg*., by surface modification.

Carbon nanotubes (CNTs) possess many attractive properties and these materials are envisioned for a broad range of applications including medical diagnosis and therapy, or a combination of both^1^. However, close attention to the potential toxicity of CNTs is warranted^2^,^3^,^4^. Previous studies in experimental animals have shown that pristine (non-functionalized) CNTs, both single- and multi-walled, can trigger pulmonary inflammation and fibrosis^5^,^6^ as well as genotoxic and carcinogenic effects in the lungs^7^,^8^. Direct exposure of humans to CNTs via the blood is not likely except in a medical setting as in the case of imaging or drug delivery^9^; however, nanomaterials could potentially translocate across organ barriers following entry into the body via other routes, upon inhalation, or following dermal and gastrointestinal exposure^10^. Nanomaterials that enter into the blood stream are known to preferentially accumulate in the hepatic region and also in the spleen, two organs rich in reticuloendothelial (phagocytic) cells. For instance, using a new, label-free mass spectrometry-based method, Chen *et al*. could map the sub-organ distribution of carbon-based nanomaterials including CNTs in mice; accumulation was evidenced in reticuloendothelial system enriched tissues such as lung and liver^11^.

It has been demonstrated that the adsorption of proteins and other biomolecules onto the surface of nanomaterials determines their cellular uptake and modulates their toxicity^12^. Using theoretical and experimental approaches, Ge *et al*. demonstrated that SWCNTs adsorbed serum proteins in a competitive manner, and that the corona of proteins mitigated the cytotoxicity of SWCNTs^13^. The protein corona has been shown to form rapidly (within less than a minute) and to affect hematocompatibility, *i.e.*, thrombocyte activation, hemolysis, endothelial cell death of silica and polystyrene nanoparticles^14^. In nanomedicine, strategies to avoid adsorption of proteins and thereby reduce the non-specific uptake by the reticuloendothelial system are commonly applied. These include coating or covalent attachment of polymers such as polyethylene glycol (PEG) onto the nanomaterial. PEGylated SWCNTs exhibit relatively long blood circulation times and reduced uptake by the reticuloendothelial system, allowing for efficient tumor targeting in mice^15^ with excretion and clearance of the PEGylated SWCNTs via the biliary and renal pathways^16^. Other studies have confirmed that functionalized CNTs can exit from the systemic blood circulation through renal excretion^17^,^18^. Indeed, functionalized CNTs may well be exploited as versatile delivery systems in nanomedicine provided that their degradation and/or clearance can be controlled^19^. However, accumulation of CNTs in the hepatic region following intraperitoneal or intravenous injection remains a potential concern^20^,^21^. The biodistribution of functionalized SWCNTs was studied by Raman spectroscopy by Liu *et al*. who observed dominant uptake in the liver and spleen over other organs and tissues^16^. Similarly, Yang *et al*. investigated the blood circulation time and *in vivo* distribution of PEGylated, ^13^C-enriched SWCNTs in mice and noted preferential trapping in the liver^22^.

Notably, the liver is a key organ involved in metabolism, detoxification, synthesis of proteins and lipids, secretion of cytokines and growth factors and immune/inflammatory responses. The cytochrome P450 (CYP450) enzymes are a diverse group of proteins which are responsible for the initial biotransformation of xenobiotic compounds and drug metabolism; in addition, many substances (prodrugs) are bioactivated by CYPs to form their active compounds^23^. Previous studies have shown that silver nanoparticles and polystyrene nanoparticles can affect the enzymatic function of CYPs^24^,^25^,^26^. However, no data are available on the potential impact of CNTs on CYP activity. In this study, we turned our attention to CYP3A4, the most prominent cytochrome P450 isoenzyme^27^. Using a combination of computational and experimental approaches, we find that carboxylated SWCNTs (c-SWCNTs) inhibit CYP3A4 in a dose-dependent manner. This has important implications for *in vivo* applications involving CNTs, as we shall discuss below.

## Results and Discussion

### c-SWCNTs dose-dependently inhibit CYP3A4

**Figure 1:**
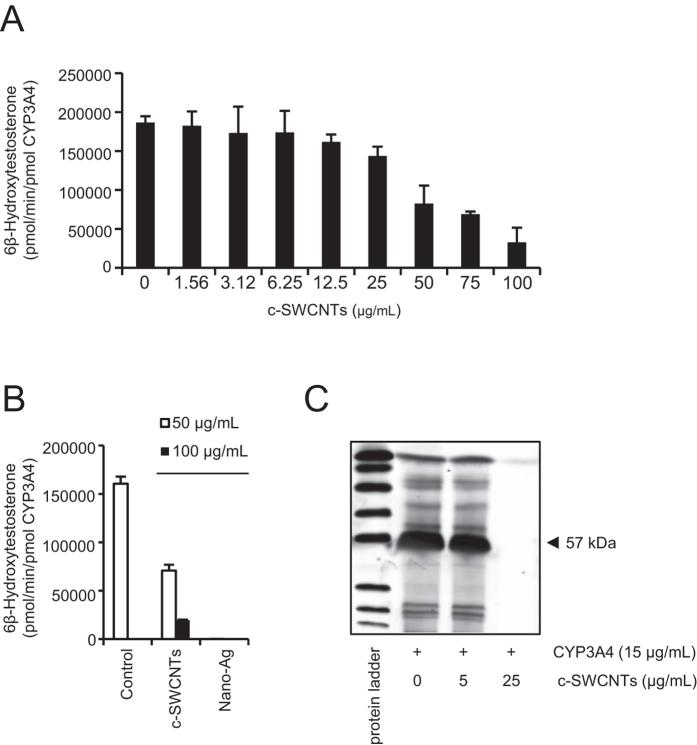
Carboxylated SWCNTs dose-dependently inhibit CYP3A4. (**A**) The conversion of testosterone, to 6β-hydroxy testosterone as a measurement of the enzymatic activity of CYP3A4 was determined by HPLC in the presence of the indicated amounts of c-SWCNTs. (**B**) Citrate-coated silver nanoparticles also inhibit the enzymatic activity of CYP3A4. Data are shown as mean values ± S.D. (**C**) Interaction between CYP3A4 protein and c-SWCNTs. Supernatants were taken after incubation of recombinant human CYP3A4 and c-SWCNTs at the indicated concentrations for 60 min and analyzed by SDS-PAGE. The results show that the CYP3A protein is adsorbed by c-SWCNTs (25 µg/mL).

In the present study, SWCNTs synthesized by chemical vapor deposition (CVD) were subjected to oxidation that resulted in the generation of oxygen-containing functional groups on the surface of the SWCNTs. This yielded short, carboxylated SWCNTs (c-SWCNTs) (Supplementary Information, Table S1). The impact of c-SWCNTs on CYP450 function was assessed using commercially available bactosomes, *i.e.*, human hepatic cytochrome P450 isoenzymes (here: CYP3A4) coexpressed functionally in *Escherichia coli* with human NADPH-P450 reductase. *E. coli*-expressed CYPs have been validated as surrogates to their counterparts in human liver microsomes^28^ and previous studies suggested that such recombinant enzymes may be suitable for human metabolism studies^29^. Although various fluorescence-based assays are available for measuring the activity of CYPs, and such assays have been used previously to record effects of non-metallic nanoparticles^26^, carbon-based nanomaterials are known to interfere with many commonly used dye-based assays^30^. Therefore, we utilized high performance liquid chromatography (HPLC) to measure the conversion of testosterone to 6β-hydroxy testosterone, as an indicator of CYP3A4 activity. c-SWCNTs were incubated with CYP3A4-containing bactosomes in the presence of the NADPH regenerating system. Our results demonstrated that there is a dose-dependent inhibition of CYP3A4 (Fig. 1A). Silver nanoparticles were previously shown to inhibit CYPs, in particular CYP3A4^25^. Indeed, citrate-coated silver nanoparticles suppressed the conversion of testosterone to 6β-hydroxy testosterone, at doses of 50 or 100 μg/mL, thus acting as a benchmark in the present study (Fig. 1B). Additionally, to provide evidence for a direct interaction between CYP3A4 and c-SWCNTs, we performed SDS-PAGE experiments. To this end, 15 μg/mL of recombinant human CYP3A4 protein were incubated with c-SWCNTs at 5 or 25 μg/mL. Samples were then centrifuged and the free protein in the supernatants was subjected to SDS-PAGE, as described previously for serum proteins adsorbing to SWCNTs^13^. The results showed the adsorption of CYP3A4 proteins by c-SWCNTs at 25 μg/mL, but not at 5 μg/mL (Fig. 1C).

**Figure 2:**
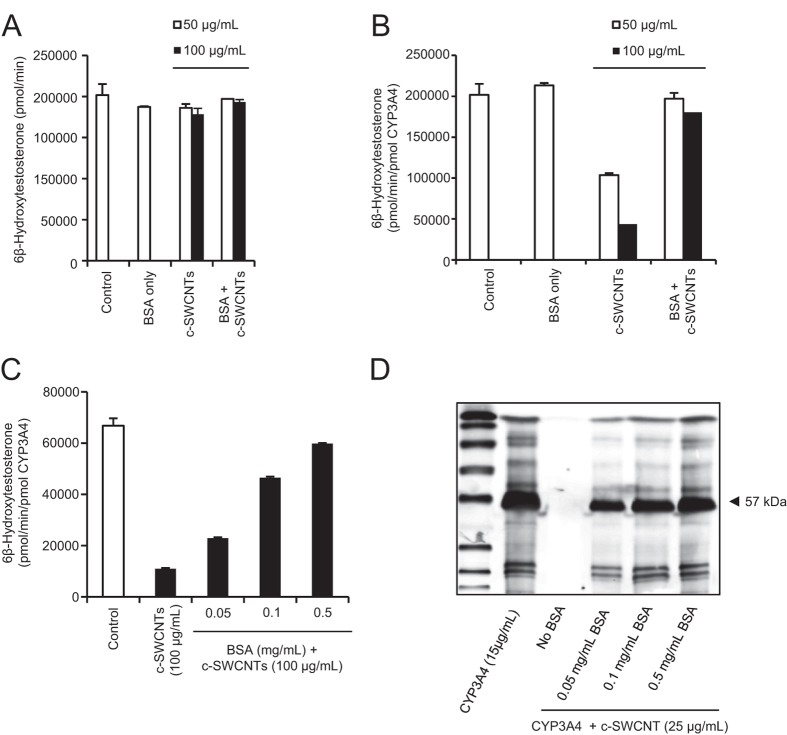
Mitigation of CYP3A4 inhibition by a protein corona. (**A**) The addition of c-SWCNTs or c-SWCNTs with a corona of bovine serum albumin (BSA) to the reaction mixture after completion of the enzymatic reaction demonstrated that c-SWCNTs do not interfere with the assay (*i.e.*, no evidence of adsorption of the metabolite, 6β-hydroxy testosterone). (**B**) The effect of bovine serum albumin (BSA) adsorbed onto c-SWCNTs on the enzymatic activity of CYP3A4. (**C**) The protein corona effect is dependent upon the amount of BSA adsorbed onto the c-SWCNTs. BSA was quantified using the BCA assay. Conversion of testosterone, to 6β-hydroxy testosterone was determined by HPLC. Data are shown as mean values ± S.D. (**D**) SDS-PAGE analysis shows that the recombinant human CYP3A4 protein is adsorbed by c-SWCNTs following 60 min incubation and that this interaction is prevented by BSA in a concentration-dependent manner.

Xia *et al*. predicted and provided experimental evidence for the adsorption of small molecules, as well as steroid hormones, onto multi-walled CNTs^31^, and this observation led us to question whether the observed inhibition of CYP3A4 conversion of testosterone to 6β-hydroxy testosterone could be explained by adsorption of the parent compound or its metabolites. To rule out this potential artifact, we performed a simple experiment in which CYP3A4 activity was evaluated with or without the addition of c-SWCNTs to the solution after the enzymatic reaction was run to completion. Our results showed that the 6β-hydroxy testosterone concentration, determined by HPLC, was similar to that of the control (without c-SWCNTs) (Fig. 2A). This, therefore, validates our observation that c-SWCNTs do not interact with the metabolites, under the present experimental conditions.

### Protein corona prevents inhibition of CYP3A4

Albumin was identified as the major fetal bovine or human serum/plasma protein adsorbed onto SWCNTs and albumin coating was shown to modulate the effects of SWCNTs in a murine macrophage cell line^32^. To assess the potential role of the protein corona on the inhibition of CYP3A4 activity, we pre-coated the c-SWCNTs with bovine serum albumin (BSA). BSA has an isoelectric point of ~5.64 giving it a high affinity for physical adsorption onto c-SWCNTs. Measurements of the zeta potential of c-SWCNTs with/without a corona of BSA revealed a negative surface charge (Supplementary Information, Table S1). Experiments in which CYP3A4-containing bactosomes were incubated with c-SWCNTs with/without a pre-formed corona clearly demonstrated that the protein corona prevented the enzyme inhibition by the c-SWCNTs (Fig. 2B). BSA alone had no effect on the activity of CYP3A4, as determined by HPLC. The amount of BSA on the c-SWCNTs was measured by means of the BCA protein assay. As shown in Fig. 2C, the effect was related to the amount of protein in the corona. To further validate the inhibitory effects of the albumin corona present on the surface of the c-SWCNTs on the adsorption of CYP3A4 proteins, we performed SDS-PAGE experiments using recombinant human CYP3A4 protein and c-SWCNTs with/without pre-treatment with different concentrations of BSA (0.05 to 0.5 mg/mL). The free protein in the supernatants was subjected to SDS-PAGE and we observed a significant reduction in the adsorption of the CYP3A4 proteins on the surface of the c-SWCNTs that appeared to be proportional to the amount of BSA (Fig. 2D). In the absence of BSA, the CYP3A protein remained attached to the c-SWCNTs.

### PEG-functionalization mitigates CYP3A4 inhibition

**Figure 3:**
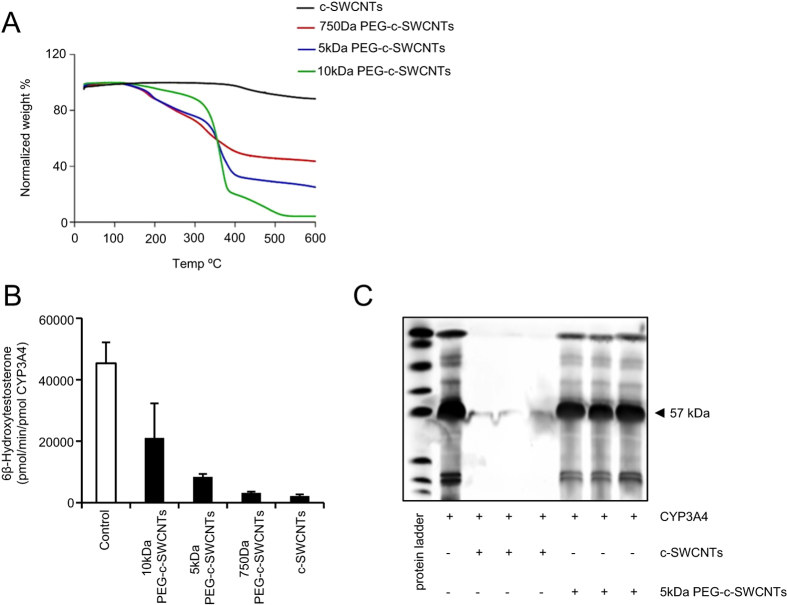
PEG-functionalization mitigates CYP3A4 inhibition. (**A**) TGA was performed to confirm the chemical conjugation of PEG onto c-SWCNTs: a higher weight loss is due to the higher MW of the decomposed chain (see Materials and Methods for details). (**B**) The effect of different molecular weight (MW) polyethylene glycol (PEG) chains (750Da, 5 kDa, 10 kDa) on the c-SWCNT-mediated inhibition of enzymatic activity of CYP3A4, as determined by HPLC-based detection of 6β-hydroxy testosterone. The c-SWCNT concentration was 100 ug/mL in all samples. The data are shown as mean values ± S.D. (**C**) Supernatants were taken after incubation of recombinant human CYP3A4 and c-SWCNTs or 5 kDa PEG-c-SWCNTs and analyzed by SDS-PAGE. Three lanes with CYP3A + c-SWCNTs and three lanes with CYP3A + 5 kDa PEG-c-SWCNTs incubated for 0, 30, or 60 min are shown; the differences between these time-points were minimal, however, indicating that the impact of PEGylation on the interaction with CYP3A4 was prompt.

Next, we examined the potential role of PEG functionalization. To this end, c-SWCNTs were covalently functionalized with linear PEG chains of various molecular weights (750 Da, 5 KDa, and 10 KDa). PEG chemical grafting was confirmed by means of TGA (Fig. 3A) and the covalent bonding of PEG to the c-SWCNTs was confirmed by FTIR (Supplementary Information, Fig. S1). PEG-functionalized c-SWCNTs retained their negative surface charge (Supplementary Information, Table S1). Our results demonstrated that PEGylation prevented the c-SWCNT-mediated inhibition of CYP3A4, possibly through repulsion between the c-SWCNTs and bactosomes. Indeed, the reconstitution of the enzymatic activity of CYP3A4 was proportional to the molecular weight of the PEG chains on the surface of the c-SWCNTs (Fig. 3B). Additionally, using SDS-PAGE, we noted a marked inhibition of CYP3A4 interactions with the surface of the 5 kDa functionalized SWCNTs when compared with the non-functionalized c-SWCNTs (Fig. 3C). Taken together, these results have suggested that PEGylation reduces the c-SWCNTs mediated inhibition of the enzymatic activity of CYP3A4 through a reduction in surface adsorption of the protein.

### Modeling of c-SWCNT-CYP3A4 interactions

The active site of CYP3A4 is located at the hydrophobic core of the protein and is connected to the surface of the enzyme through access channels; the substrates/products are presumed to enter/leave the active site by access/exit channel^33^. Indeed, employing steered MD simulations, Fishelovitch *et al*. identified six distinct conduits (named 2a, 2b, 2c, 2e, 3, and S)^33^. By computing path-dependent estimates of work during ligand egression, the authors argued that two particular channels (2e and 3) are best suited for the departure of 6β-hydroxy testosterone (the metabolite of interest in the present study). To begin to shed light on potential inhibitory mechanisms, we firstly performed MD simulations^34^ aimed at illuminating CNT-CYP3A4 interactions in the neighborhood of the major 2e egress conduit (Supplementary Information, Fig. S2a). We thus prepared four distinct simulation systems, each consisting of a c-SWCNT and a CYP3A4 molecule placed at different relative orientations. In the first system (Model 1), the c-SWCNT was configured with its sidewall facing the exit of the 2e channel (Supplementary Information, Fig. S2b). In the second system (Model 2), the CNT was positioned in an orthogonal manner with its terminal carboxyl groups (and tube opening) facing the exit of the same conduit (Supplementary Information, Fig. S3a–d). In the third system (Model 3), the CNT is simply translated downward from its Model 1 configuration such that the nanotube’s sidewall and terminal edge are both proximal to the channel of interest (Supplementary Information, Fig. S3e–h). In the fourth system (Model 4), the c-SWCNT was set up to face the 3 and S channels (see Supplementary Information, Fig. S4a for details). Simulations starting from these initial configurations were run in triplicate to time scales of at least 100 ns (Supplementary Information, Fig. S4b,c). Subsequent analysis revealed that the terminal carboxyl groups on the c-SWCNTs (initialized close to the 2e channel in Models 2 and 3) do not enter or serve to block the access channel in question. Instead, the charged, oxygen-rich functional groups appeared to preferentially bind to basic or hydrophilic protein residues situated some distance from the 2e conduit exit. Carboxyl groups may thus not be directly involved in enzymatic inhibition with respect to the 2e pathway; however, such modifications could act to guide the approach of the c-SWCNT towards CYP3A4 through long-range electrostatic attraction. For a better illustration, some intermediate states are presented in Supplementary Information, Fig. S4 showing the key interacting patterns for the charged, oxygen-rich functional groups at the end of the c-SWCNT with the basic or hydrophilic residues of the protein. As shown in Supplementary Information, Fig. S5a,b, the negatively charged carboxyl groups can form salt bridges with the positively charged residues like Lys34, Lys35, Lys251 and Lys254, and can also form hydrogen bonds with polar residues like Gln78 and Ser29. In addition, the contact ratio of carboxyl groups of the c-SWCNT with some specific basic residues (*i.e.*, Lys, Arg and His) and polar residues (*i.e.*, Gln, Ser and Asn) were added in the Supplementary Information, Table S2. From this table, one can see that a considerable proportion of specific residues in contact with carboxyl groups of the c-SWCNT can last over 50% of the simulation time, which suggests an important role of the long-range electrostatic attractions during the binding process. The role of the long-range electrostatic attractions in guiding the motion of charged nanoparticles towards a target protein or enzyme has been previously reported^35^,^36^,^37^,^38^. For example, using MD simulations, we recently found that the motion of gold nanoclusters coated with the highly charged peptides (+5e/peptide) can be tuned by the electrostatic attraction between the coating peptides and the enzyme thioredoxin reductase 1^38^. Meanwhile, other recent studies have also shown that the adsorption or immobilization of protein/enzyme can be guided by the electrostatic attractions between the negatively charged carboxyl group of graphene oxide and the positively charged residues in the protein^35^,^36^,^37^.

**Figure 4:**
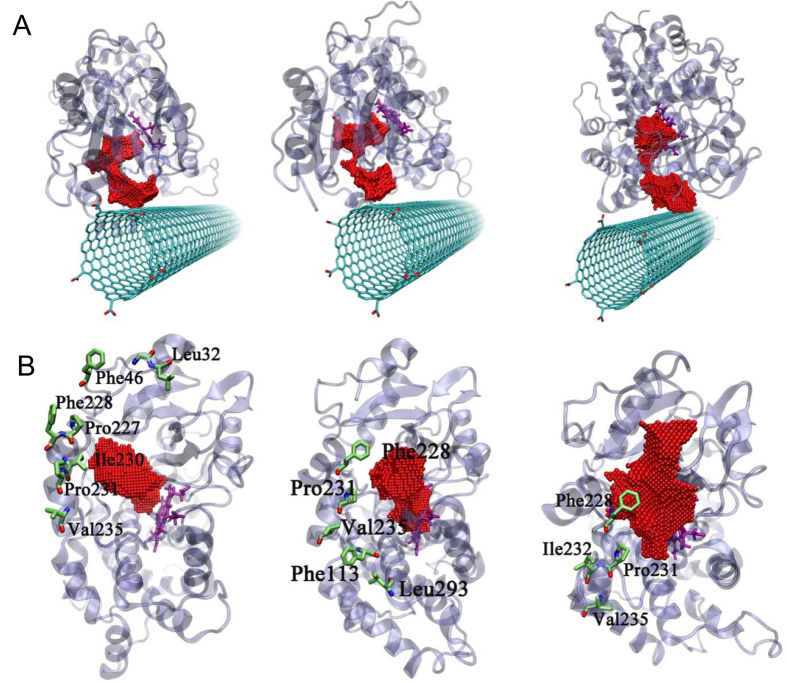
Molecular dynamics simulations suggest that c-SWCNTs can block the exit channel of CYP3A4. The last frames of three independent trajectories of Model 1 (side-by-side; consult main text) running for 120 ns (**A**). The key hydrophobic and aromatic residues of CYP3A4 binding to c-SWCNT of three trajectories (**B**). The red van der Waals (vdW) balls represent the 2e channel and the active center is shown by purple sticks.

By contrast, all three trajectories initialized from Model 1 suggested that c-SWCNTs can effectively block the 2e channel via direct sidewall binding (Fig. 4A; and see Supplementary Information, Movies M1–M4, for the two independent runs [run 1 and run 2] with both top and side views, to better capture the adsorption process). Figure 4B shows how aromatic residues belonging to CYP3A4 come into contact with the sidewall of the c-SWCNT, leading to binding stabilized by hydrophobic and π-π stacking interactions; individual π-π stacks, in general, are estimated to supply up to ~10 kcal/mol of binding free energy^39^. Formation ratio of π-π stacking interaction between some key aromatic residues, such as Phe46, Phe113, and Phe228, with the surface of c-SWCNT were calculated and presented in Supplementary Information, Table S3. As for π-π stacking interaction, there are two energetically favorable configurations, with the aromatic rings in parallel (flat, face-to-face) or perpendicular to the c-SWCNT surface (T-shape, edge-to-face)^39^. In the current case, when a packing pattern between the aromatic residues and the c-SWCNT is similar to any of the two classical configurations, a π-π stacking was considered formed. As shown in Supplementary Information, Table S3, the formation ratio of π-π stacking for all the key aromatic residues mentioned above is over 50%, which demonstrates that the π-π stackings indeed play a crucial role during the binding process. Interestingly, we also noted that the specific residues Phe228, Pro231 and Val235 featured prominently in all three Model 1 trajectories (Fig. 4B). As for the blocking time, the starting time for the 2e channel being blocked was approximately 40, 10, and 28 ns for run 1, run 2, and run 3, respectively (Supplementary Information, Fig. S6a–c). From then on, the blocked states remained unchanged until the end of the simulations. Thus, overall, at least 78.3% of the simulation times are with the 2e channel blocked.

Besides the main 2e channel, Model 4 was also set up and simulated to study the response of S channel and the relatively minor 3 channel to the presence of c-SWCNT. The S channel was previously suggested as a substrate access channel^40^. As shown in Supplementary Information, Fig. S4a, very similar to the main 2e channel, both the entrance of S channel and 3 channel were directly blocked by c-SWCNT (Supplementary Information, Fig. S4b,c). The 3 channel was found to be completely closed, which seems to be related to the allosteric effect induced by the binding of c-SWCNT.

**Figure 5:**
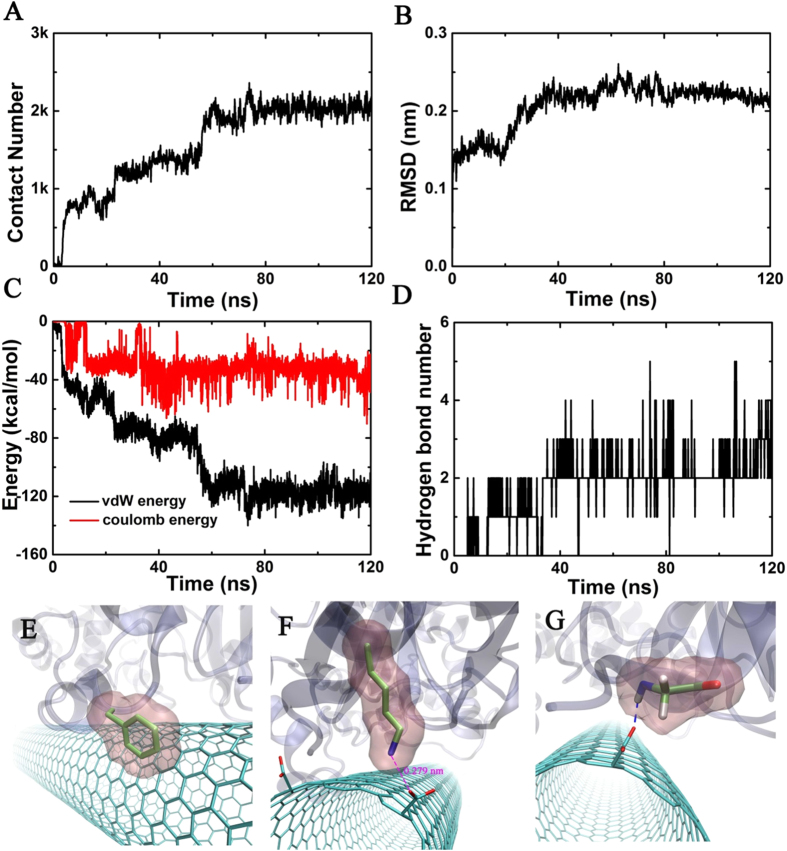
Molecular dynamics simulations reveal the binding process of c-SWCNT to CYP3A4. The heavy atom contact number (**A**), the root mean square deviation (RMSD) of alpha carbon of CYP3A4 (**B**), the van der Waals (vdW) and Coulomb interaction energy (**C**) and hydrogen bond number (**D**) between carboxylated CNT and CYP3A4 as function of time. Local snapshots showing the π-π stacking interaction (**E**), salt bridge interaction (**F**) and hydrogen binding interaction (**G**) by Phe228, Lys35 and Gly31, respectively. Key residues are shown as colored sticks and as transparent pink surfaces.

To more clearly elucidate the mechanism by which CYP3A4 binds to the c-SWCNT in Model 1, heavy atom contact numbers (between the enzyme and nanotube) and α–carbon root mean square deviations (RMSDs) (from the initial protein configuration) were calculated based on one representative trajectory (Fig. 5A,B), in which some key hydrophobic and aromatic residues were highlighted. As the plots illustrate, both the heavy atom contact number and RMSD reach steady state values fluctuating around ~2000 and ~0.2 nm, respectively, after about 80 ns of simulation time. Despite the considerable number of contacts that form between the protein and the nanotube, the enzyme’s structure deforms only slightly as the CNT associates. Thus, the data do not support catastrophic protein unfolding/misfolding of CYP3A4 that would lead to a total ablation of enzyme activity.

To better understand the physical driving forces behind the nanotube-CYP3A4 interactions, we decomposed the interactions between CYP3A4 and the c-SWCNT in terms of vdW and electrostatic energetic components and a count of hydrogen bonds (Fig. 5C,D). In the steady state regime, the prominence of dispersion and π-π stacking interactions was quite evident: the vdW interaction energy dropped below and remained near −120 kcal/mol, forming the basis for sustained binding. However, electrostatic interactions also contributed to the adsorption process, particularly during the nascent stages of approach and binding. At early time-points, electrostatic and vdW energies were nearly equal in magnitude. The number of hydrogen bonds between CYP3A4 and the c-SWCNT fluctuated, yet increased somewhat over the course of the simulation (Fig. 5D). These data show that the interaction of CYP3A4 with c-SWCNTs is sustained mainly by the dispersive components of hydrophobic, π-π stacking, and hydrogen bonding interactions. However, adsorption is driven in part by early electrostatic attraction. To illustrate specific π-π stacking, salt-bridge and hydrogen bonding interactions between CYP3A4 and the c-SWCNT, representative snapshots that capture relevant phenylalanine, lysine and glycine residues are presented in Fig. 5E–G. A phenylalanine residue (Phe228) is seen adjacent to the sidewall of the c-SWCNT (Fig. 4B, left image) forming a tight π-π stack, a dominant interaction mode noted in the adsorption of other proteins onto various carbon-based nanomaterials^34^. Meanwhile, the central and right images in Fig. 4B illustrate, respectively, how a lysine residue (Lys35) engages in a strong salt-bridge interaction with a CNT carboxyl group and a glycine (Gly31) forms a backbone hydrogen bond with separate nanotube oxygen. Overall, our simulation data suggest that a combination of different interaction types contribute to the c-SWCNT-mediated blocking of the 2e access channel to the active site of the CYP3A4 enzyme.

### AFM imaging of c-SWCNTs and bactosomes

**Figure 6:**
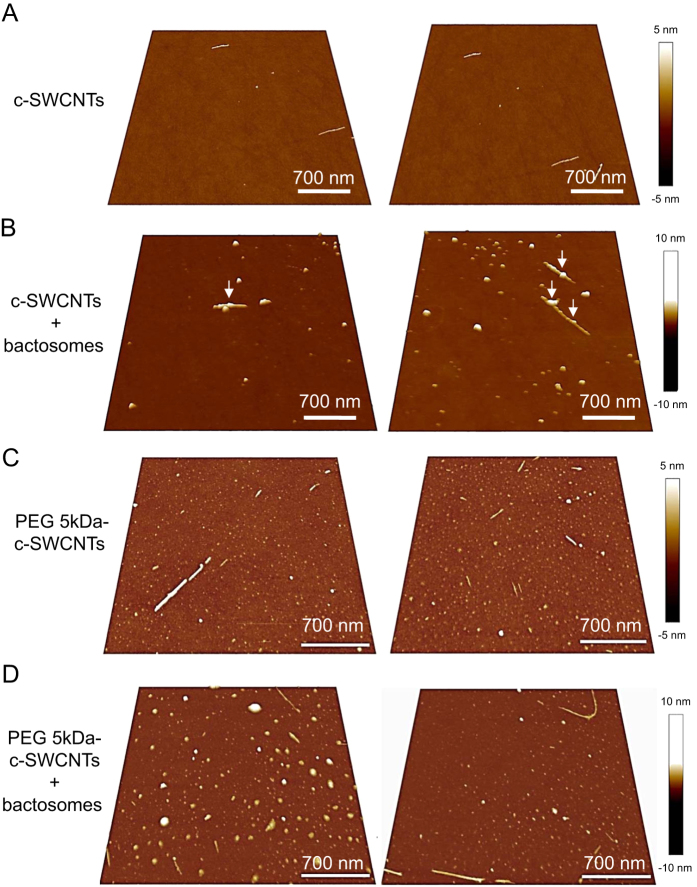
Direct interaction of c-SWCNTs and PEG 5 kDa-c-SWCNTs with CYP3A4. Atomic force microscopy (AFM) imaging of (**A**) c-SWCNTs alone, (**B**) CYP3A4-containing bactosomes incubated with c-SWCNTs, (**C**) PEG 5 kDa-c-SWCNTs alone, and (**D**) CYP3A4-containing bactosomes incubated with PEG 5 kDa-c-SWCNTs, suggested that the uncoated c-SWCNTs interact with CYP3A4 via side-walls while the PEG 5 kDa coating on the surface of the c-SWCNTs significantly reduces this interaction (and see Supplementary Information, Fig. S7). AFM images were acquired in a non-contact mode with large scale scans of more than 1 μm^2^. At least three random areas per condition were scanned.

AFM has been widely applied to monitor interactions between biomolecules and nanomaterials including CNTs^13^. In bactosome-free images, we noted that long, isolated CNTs and 5 kDa PEG functionalized c-SWCNTs were strewn across the silicon substrate surface (Fig. 6A,C). When bactosomes were introduced into the system, few bare nanotubes appeared in our micrographs: uncoated c-SWCNTs seemed to readily attach to CYP3A4-containing bactosomes via their sidewalls (93 ± 27%) (Fig. 6B) as compared to the PEG 5 kDa-c-SWCNTs where the PEG coating provided “stealth” to the c-SWCNTs significantly reducing the attachment of CYP3A4-containing bactosomes on their sidewalls (14.2 ± 4%) (Fig. 6D). Differences in the attachment of CYP3A4-containing bactosomes on the sidewalls of uncoated c-SWCNTs and PEG 5 kDa-c-SWCNTs were found to be statistically significant (P < 0.001) (Supplementary Information, Fig. S7). Thus, the AFM images not only provide verification of the theoretical simulations, but also showed the interference induced by the surface functionalization of the c-SWCNTs. While our AFM images demonstrated that bactosomes directly interact with CNTs in a side-on fashion, specific interactions between c-SWNCTs and CYP3A4 obviously cannot be resolved using this approach. The MD simulations, on the other hand, provided a means of linking the experimental findings to mechanisms of association between CNTs and the CYP3A4 enzyme.

## Conclusions

Cytochrome P450 enzymes are responsible for the metabolism of thousands of endogenous and xenobiotic substrates, including drugs. CYP3A4, in particular, is of paramount importance, because it is the most abundant P450 in the human liver and is known to metabolize the majority of drugs for which the pathway of biotransformation is known^27^. As nanomaterials, including CNTs, frequently find their way to the liver following parenteral administration, despite attempts to avoid uptake by the reticuloendothelial system through surface functionalization of the nanomaterials, it is critical to understand the potential effects on liver function, not least on the detoxification and elimination of drugs and other xenobiotics through the cytochrome P450 enzymatic system.

We found in the present study that c-SWCNTs can interfere with human CYP3A4 function, focusing on the metabolism of testosterone to 6β-hydroxy testosterone. Using SDS-PAGE and AFM, we provided evidence for a direct interaction between CNTs and CYP3A4. MD simulations showed that c-SWCNTs can effectively block a particular molecular access conduit (access channel 2e) that leads to the catalytic center of CYP3A4. Overall, through this computational approach, our results confirmed that the c-SWCNTs can adsorb onto the exit of the 2e channel of CYP3A4 through a complex binding mechanism, with hydrophobic, π-π stacking and vdW interactions playing a dominant role, while the Coulomb and hydrogen bond interactions also promoted this interaction. Previous work identified two potential routes for CNTs to inhibit the function of proteins, one through the disruption (“plugging”) of the active site^41^, and the other through competitive binding of incoming ligands^42^. Here, we have potentially uncovered a third route of inhibition related to blocking access to/dissociation from the active site of an enzyme (CYP3A4). Notably, the formation of a protein corona on the c-SWCNTs mitigated the observed inhibition of CP3A4, as did PEGylation, probably due to the weakened hydrophobic interaction with CYP3A4 due to pre-adsorbed BSA in the former case and the unfavorable steric effect in the latter case. One may ask whether the current observations are relevant for the *in vivo* (clinical) situation. Indeed, while several pre-clinical studies have shown that CNTs may accumulate in the hepatic region, it is pertinent to ask whether relevant concentrations of these nanomaterials are achieved *in vivo* in the liver. Overall, there is a paucity of data on the actual concentrations of nanomaterials *in vivo* not only in the liver as a whole, but also the concentrations in specific cell populations in the liver. Nevertheless, in studies using functionalized, isotope-labeled SWCNTs, in which mice were intravenously exposed to PEG-SWCNTs at a single dose of 2.4 mg SWCNTs equivalent/kg body weight, or 60 μg SWCNTs equivalent in 200 mL, the amounts of SWCNTs accumulating in various tissues could be determined^22^. Hence, the authors noted that the hepatic accumulation level was 19.1%ID/g (percentage of injected dose per gram) for the PEGylated SWCNTs, while 25.9%ID/g remained in the spleen, when measured 7 days post exposure. Moreover, using Raman spectroscopy to monitor the long-term fate of functionalized SWNCTs in mice, Dai and co-workers were able to show that appreciable amounts of 2 kDa PEG-SWCNTs remained in the liver even at 3 months post-exposure with a concentration of approximately 7%ID/g, while lower levels (approximately 2%ID/g) of 5 kDa PEG-SWCNTs were retained at 3 months^16^. The initial injected dose in the latter study was 200 μL of 0.1 mg/mL SWCNTs solution. Thus, it appears that the concentrations used in the present study (0 to 100 μg/mL) are not unrealistic when compared to the amounts of CNTs that have been found to accumulate in the hepatic region in mice upon i.v. injection^16^,^22^. However, it is important to point out that the present *in vitro* and modeling study was a proof-of-principle study, assessing whether CNTs may inhibit CYP450s. The potential link to the clinical situation is obviously of considerable importance and we hope that this work will inspire further studies. Notably, under *in vivo* conditions, when administered into the blood stream, CNTs would be coated with a bio-corona of proteins and other biomolecules, a process that is believed to occur very rapidly^14^. This, in turn, is likely to impact on their biocompatibility and cellular uptake. However, while PEGylation or other surface modifications may reduce non-specific protein adsorption, thus affording “stealth” to nanomaterials, it is noted that corona formation is not entirely prevented by PEGylation^43^. Moreover, recent studies have shown that the protein corona may undergo degradation upon cellular uptake and trafficking of nanoparticles to the lysosomal compartment, and internalized nanoparticles could therefore interact with their biological surroundings as a function of their pristine surfaces^44^. While the present results have demonstrated that SWCNTs may inhibit CYP3A4, our data also suggest that this inhibition of enzyme activity is mitigated in the presence of a protein corona. This, therefore, suggests that in a clinical setting, when SWCNTs are cloaked in a bio-corona they should not pose a risk of disturbing xenobiotic metabolism, provided the corona is stable. However, further studies are needed to determine whether CNTs, when administered *in vivo* in a clinical setting, could impact negatively on the metabolism of endogenous or xenobiotic compounds in the liver; indeed, this deserves particular attention if CNTs are used to deliver drugs that are either metabolized or bioactivated by the cytochrome P450 enzymes. CYP3A4, the CYP450 enzyme in focus here, is responsible for the biotransformation of several important anti-cancer drugs, such as paclitaxel, doxorubicin, and docetaxel^45^. On the other hand, our results also show that PEGylation may circumvent this problem. Indeed, PEGylation of CNTs affects protein interactions which, in turn, influence the pharmacokinetic profile of these nanomaterials^43^. Overall, this study provides an example of nano-scale toxicity resulting from direct interference with biological systems – here, the enzymatic activity of CYP3A4 – but the data presented here also point towards effective strategies for mitigating this effect.

## Materials and Methods

### Nanomaterials

Pristine SWCNTs synthesized via chemical vapor deposition (CVD) technique (purity ≥95%; length 1–5 μm) were purchased from NanoLab Inc., Waltham, MA (batch number 102910) (Supplementary Information, Table S1). Citrate-coated BioPure™ Ag NPs (40 nm) were purchased from NanoComposix Inc., San Diego, CA. For PEG functionalization of c-SWCNTs, methyl-terminated linear 750, 5 kDa or 10 kDa PEG were purchased from Rapp Polymere GmbH, Tuebingen, Germany.

### Oxidation of SWCNTs

For oxidation, 120 mg of pristine SWCNTs were dispersed in a 3:1 ratio mixture of H_2_SO_4_:HNO_3_ (25 mL) using a tip-probe sonicator (5 × 9 s). The mixture was then refluxed at 110 °C for 2 h in a round bottom flask equipped with a condenser and subsequently placed in ice until the temperature reached 15 °C. The SWCNT mixture was then diluted with deionized water (50 mL) and filtered through polytetrafluoroethylene (PTFE) hydrophilic membrane discs (100 nm pore size). Samples were washed until the filtrate reached pH of deionized water and then dried in a vacuum at 80 °C overnight. Approximately 70 mg of c-SWCNTs were collected and re-suspended in milli-Q water (1 mg/mL) using a bath sonication (Branson 2510) for 35 min. Coating of the c-SWCNTs with bovine serum albumin BSA (Sigma-Aldrich) in milli-Q water was done for 20 min. The bicinchoninic acid (BCA) protein assay (ThermoScientific, Stockholm, Sweden) was used to determine the amount of adsorbed BSA.

### PEG functionalization of c-SWCNTs

Oven-dried c-SWCNTs (2 mg) were mixed with methyl-terminated linear 750 Da, 5 kDa, and 10 kDa PEG, respectively in the presence of dichloromethane (DCM) (10 mL), 4-dimethylaminopyridine (60 mg) and 1 M *N,N’*-dicyclohexylcarbodiimide (130 μL) (all reagents purchased from Sigma-Aldrich, Stockholm, Sweden). The amidation reaction was carried out for 72 h after which DCM was vaporized using a rotary evaporator. Unattached PEG molecules were removed from the samples by suspending them in milli-Q water and centrifuging them through Amicon^®^ Ultra-4centrifugal filter devices (MW: 100 kDa) (Sigma-Aldrich). Successful PEGylation of c-SWCNTs was confirmed using TGA, as described in the following section.

### Physico-chemical characterization

High Resolution Transmission Electron Microscope (HR-TEM) JEOL JEM 2100F (JEOL AB, Sollentuna, Sweden) was used for imaging c-SWCNTs. Samples were diluted in isopropanol and sonicated in a water bath to ensure proper dispersion. A few microlitres were drop-casted onto a copper TEM grid placed on filter paper and left to dry for 4 h. The TEM images were used to calculate the average length of the c-SWCNTs (Supplementary Information, Table S1). Hydrodynamic diameter (of the AgNPs) and surface charge was determined by means of dynamic light scattering (DLS) technique using a Zetasizer NanoZS (Malvern, UK) at 25 °C using 0.1 mg/mL dispersions in (18.2MΩ cm) milli-Q H_2_O. Measurements were performed 10 times, with a minimum of 10 repeats each time (Supplementary Information, Table S1). The hydrodynamic diameter of the citrate-coated AgNPs in water was found to be 40.46 ± 0.39 nm and the zeta potential was −41.3 ± 1.84 mV. Thermogravimetric analysis (TGA) (TA Q500, TA Instruments, Newcastle, DE) was used to confirm the grafting of PEG molecules on the c-SWCNTs. TGA was performed from ambient temperature up to 700 ^o^C in a nitrogen atmosphere (20 mL/min) as described^46^. TGA analysis indicated that the pure PEG decomposition temperature is between 370–420 ^o^C; and that the chemical conjugation of PEG to c-SWCNTs was successful and PEG is ~50%, ~30%, and ~24%, respectively, of the total sample weight of 750Da, 5 kDa, and 10 kDa PEG-c-SWCNTs. The ratio of PEG grafted onto the surface of the c-SWCNTs was calculated as shown in Equation (1):
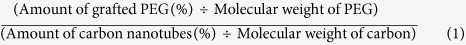
In this way, the ratio of PEG chains grafted on the surface of c-SWCNTs was found to be 1:82, 1:310, and 1:263 for the 750 Da, 5 kDa, and 10 kDa PEG-c-SWCNTs, respectively. To confirm the covalent bonding of the PEG molecules to the surface of the c-SWCNTs, Fourier Transform Infrared (FTIR) analysis was performed on the 5 kDa PEG-c-SWCNTs suspended in water using the Thermo Scientific Nicolet iS10 spectrometer in the attenuated total reflection (ATR) mode (Supplementary Information, Fig. S1).

### CYP3A4-containing bactosomes

Bactosomes (CYP3A4BR) expressing recombinant human CYP3A4 protein, NADPH-P450 reductase and cytochrome b5, and bactosomes with no recombinant enzymes as negative controls were purchased from Cypex Ltd., Dundee, UK.

### HPLC for analysis of testosterone conversion

For the study, a CYP3A4-premix containing 200 mM phosphate buffer (pH 7.4), 100 mM magnesium chloride, 20 mM testosterone, 25 pmol of CYP3A4 bactosomes was prepared. 140 μl of the CYP3A4-premix was incubated with the indicated amounts of c-SWCNTs, or BSA-coated c-SWCNTs, or 750Da, 5 kDa and 10k Da PEG-c-SWCNTs. As a negative control, CYP3A4-premix without c-SWCNTs was used and as a positive control the premix was treated with citrate-coated Ag NPs (50 or 100 μg/mL). For initiating the reaction, 40 μl of NADPH-generating system (25 mM glucose-6-phosphate, 5 U/ml glucose-6-phosphate dehydrogenase, 50 mM potassium phosphate pH 7.4, 5 mM NADP) was added to the c-SWCNTs and CYP3A4 premix suspension. The complete suspension containing the c-SWCNTs, CYP3A4-premix and NADPH-generating system were incubated at 37 °C and conversion of testosterone to 6-βhydroxyl testosterone was measured using the Waters UV-HPLC (Saint-Quentin En Yvelines, France) with a separation module (2690) and photodiode detector (2996). The mobile phase consisted of methanol (solvent A), 0.05% orthophosphoric acid (solvent B). Chromatographic separation of metabolites was done using a Kinetex™ 5 μm C18 reverse phase column (Phenomenex, Værløse, Denmark) with the mobile phase delivered at a flow rate of 1 mL/min and set at a temperature of 40 °C. Analysis was done with an isocratic elution of solvent A (56%) and solvent B (44%) for 35 min and 6-βhydroxyl testosterone was detected by UV-absorption at 240 nm.

### SDS-polyacrylamide gel electrophoresis

To assess for direct interaction of the CNTs with CYP3A4, recombinant human CYP3A4 protein and rabbit anti-human CYP3A4 antibody were purchased from Cypex Ltd. The following sets of experiments were then conducted: i) To study the quantitative effect of the concentration of c-SWCNTs on the adsorption of recombinant human CYP3A4 protein, 5 μg/mL and 25 μg/mL of c-SWCNTs were suspended with 15 μg/mL of recombinant human CYP3A4 protein. As a negative control, equal volume of phosphate buffer saline solution was added to the CYP3A4 proteins. All the suspensions were incubated at room temperature for 60 min and then the samples were centrifuged at 10,000 rpm for 10 min. The supernatants were collected and stored until further analysis by SDS-PAGE. ii) To study the inhibition of CYP3A4 protein adsorption on the surface of the c-SWCNTs by the presence of protein corona of BSA, the BSA coated (0.05, 0.1 and 0.5 mg/mL BSA), and uncoated c-SWCNTs (25 μg/mL) were mixed with recombinant human CYP3A4 protein (15 μg/mL) and samples were processed as indicated above. iii) Finally, to assess the impact of surface modification with PEG, 5 kDa PEGylated c-SWCNTs and uncoated c-SWCNTs (25 μg/mL) were incubated with recombinant human CYP3A4 protein (15 μg/mL) at room temperature for the indicted time-points. As a negative control, equal volume of phosphate buffer saline solution was added to the CYP3A4 proteins. The samples were processed as indicated above. For SDS-PAGE, equal amounts of supernatants were loaded onto a 4–12% SDS-PAGE gel and transferred to polyvinylidene difluoride membranes (Bio-Rad laboratories, Hercules, CA). The membranes were incubated with Odyssey blocking buffer purchased from Li-COR (Lincoln, NE) and probed with rabbit anti-human CYP3A4 antibody. Following incubation with horseradish peroxidase-conjugated anti-mouse secondary antibody goat anti-rabbit IRDye 800CW (Li-COR), the bound antibody was visualized with Odyssey CLx infrared based western blot analysis system and images were processed and analyzed using the Image studio version 3.1 (Li-COR).

### Atomic force microscopy

For AFM, approximately 10 μL of the ox-SWCNTs or 5 kDa PEGylated c-SWCNTs with/without CYP-expressing bactosomes suspended in sterile distilled water were dropped onto the Si wafer and dried under gentle nitrogen stream. Si wafers were first cleaned using a standard RCA process. For image acquisition, PSIA XE 150 SPM/AFM instrument (Park Systems, Suwon, Korea) was used in a non-contact mode. Topographical images were acquired using large scale scans of more than 1 μm^2^ and the images were analyzed using XEI software (Park Systems). A minimum of 3 images were acquired per condition and 20 events (or more) were analyzed per sample.

### Molecular dynamics simulation methods

Model SWCNTs were constructed with a (12, 12) wrapping topology to produce tubes with lengths of 7.0 nm (1340 carbon atoms) and diameters of 1.6 nm featuring armchair configurations. All coordinates for SWCNTs were generated using VMD (Visual Molecular Dynamics), and all CNT carbon atoms were assigned uncharged Lennard-Jones parameters of ε_cc_ = 0.36 kJ/mol and σ_cc_ = 3.4 Å. The c-SWCNTs included 11 carboxyl groups to mimic the relatively low carboxyl group concentration, with a total of 11 negative charges, mostly located at the two edges of the c-SWCNT. The crystal structure of human microsomal CYP3A4 (PDB ID: 1TQN) was used to initialize all protein configurations. Model 1, 2, 3, and 4 each contained one c-SWCNT and one CYP3A4 molecule placed in different initial configurations; the systems included 68.131, 78.748 and 100.969, and 98.596 atoms, respectively. All simulations were carried out on a Linux cluster using the software GROMACS (version 4.6.6) with the CHARMM 27 force field. The v-rescale thermostat and Parrinello-Rahman pressure coupling scheme were used to set the temperature and pressure at constant to 300K and 1 bar, respectively. Particle Mesh Ewald (PME) was employed to compute long-range electrostatic interactions and the cut-off for treating the van der Waals interactions was set to 10 Å. The TIP3P water model was used in all simulations and the c-SWCNT was constrained during the whole trajectory.

## Additional Information

**How to cite this article**: El-Sayed, R. *et al*. Single-Walled Carbon Nanotubes Inhibit the Cytochrome P450 Enzyme, CYP3A4. *Sci. Rep.* **6**, 21316; doi: 10.1038/srep21316 (2016).

## Supplementary Material

Supplementary Information

Supplementary Video S1

Supplementary Video S2

Supplementary Video S3

Supplementary Video S4
