# Supplementary material for: Single-Walled Carbon Nanotubes Inhibit the Cytochrome P450 Enzyme, CYP3A4
Source: Sci Rep. 2016 Feb 22;6:21316. doi: 10.1038/srep21316 (PMC4761960; doi:10.1038/srep21316)
Supplement: Supplementary Information [file srep21316-s1.pdf]

Supporting information

Single-Walled Carbon Nanotubes Inhibit the Cytochrome P450 Enzyme, CYP3A4

Ramy El-Sayed,[1] Kunal Bhattacharya,[1] Zonglin Gu,[2] Zaixing Yang,[2] Jeffrey K. Weber,[3] Hu Li,[4] Klaus Leifer,[4] Yichen Zhao,[5] Muhammet S. Toprak,[5] Ruhong Zhou, [2,3,6] and Bengt Fadeel[1,7]

[1]Nanosafety & Nanomedicine Laboratory, Division of Molecular Toxicology, Institute of Environmental Medicine, Karolinska Institutet, 17177 Stockholm, Sweden; [2]Institute of Quantitative Biology and Medicine, Collaborative Innovation Center of Radiation Medicine of Jiangsu Higher Education Institutions, Soochow University, Suzhou 215123, China; [3]IBM Thomas J. Watson Research Center, Yorktown Heights, New York 10598, USA; [4]Department of Engineering Sciences, Uppsala University, 75121 Uppsala, Sweden; [5]Functional Materials Division, Department of Materials and Nanophysics, KTH-Royal Institute of Technology, 16440 Stockholm, Sweden; [6]Department of Chemistry, Columbia University, New York, New York 10027, USA; [7]Department of Environmental and Occupational Health, University of Pittsburgh, Pittsburgh, Pennsylvania 15219, USA. **Correspondence:** bengt.fadeel@ki.se; or ruhongz@us.ibm.com.

Supplementary Table 1 – physicochemical characterization of the SWCNTs.

	Length (nm)	Zeta potential (mV)
c-SWCNTs	179 ± 100	-61.2
BSA@c-SWCNTs		-16.0
PEG750@c-SWCNTs		-46.5
PEG5K@c-SWCNTs		-50.2
PEG10K@c-SWCNTs		-36.2

Size was determined based on TEM images (n=100). Zeta potential of CNTs at 25°C in milli-Q H_2O.

Supplementary Table 2.

Model		Contacting residues and contact ratio
2	run1	Lys35 (75.10%), Asn104 (19.30%), Lys378 (45.80%), Lys379 (52.50%)
	run2	Ser29 (81.90%), Lys34 (64.20%), Lys35 (67.90%), Gln78 (78.20%)
	run3	His28 (33.10%), Lys35 (81.70%), Lys390 (57.00%)
3	run1	Lys251 (90.41%), Lys254 (79.04%)
	run2	Arg243 (25.30%), Lys251 (48.90%)
	run3	His28 (90.80%), Ser29 (62.70%), Lys35 (47.80%)

Supplementary Table 3.

Model 1	Contacting residues and formation of π–π stacking (time ratio)
run 1	Phe46 (69.92%), Phe228 (54.92%)
run 2	Phe113 (86.50%), Phe228 (50.50%)
run 3	Phe228 (96.67%)

Supplementary Movies

Movie M1. MD simulation of Model 1 (consult main text). The animation shows an independent run [run 1, side view] of the adsorption process of CYP3A4 to the surface of c-SWCNT.

Movie M2. MD simulation of Model 1 (consult main text). The animation shows an independent run [run 1, top view] of the adsorption process of CYP3A4 to the surface of c-SWCNT.

Movie M3. MD simulation of Model 1 (consult main text). The animation shows an independent run [run 2, side view] of the adsorption process of CYP3A4 to the surface of c-SWCNT.

Movie M4. MD simulation of Model 1 (consult main text). The animation shows an independent run [run 2, top view] of the adsorption process of CYP3A4 to the surface of c-SWCNT.

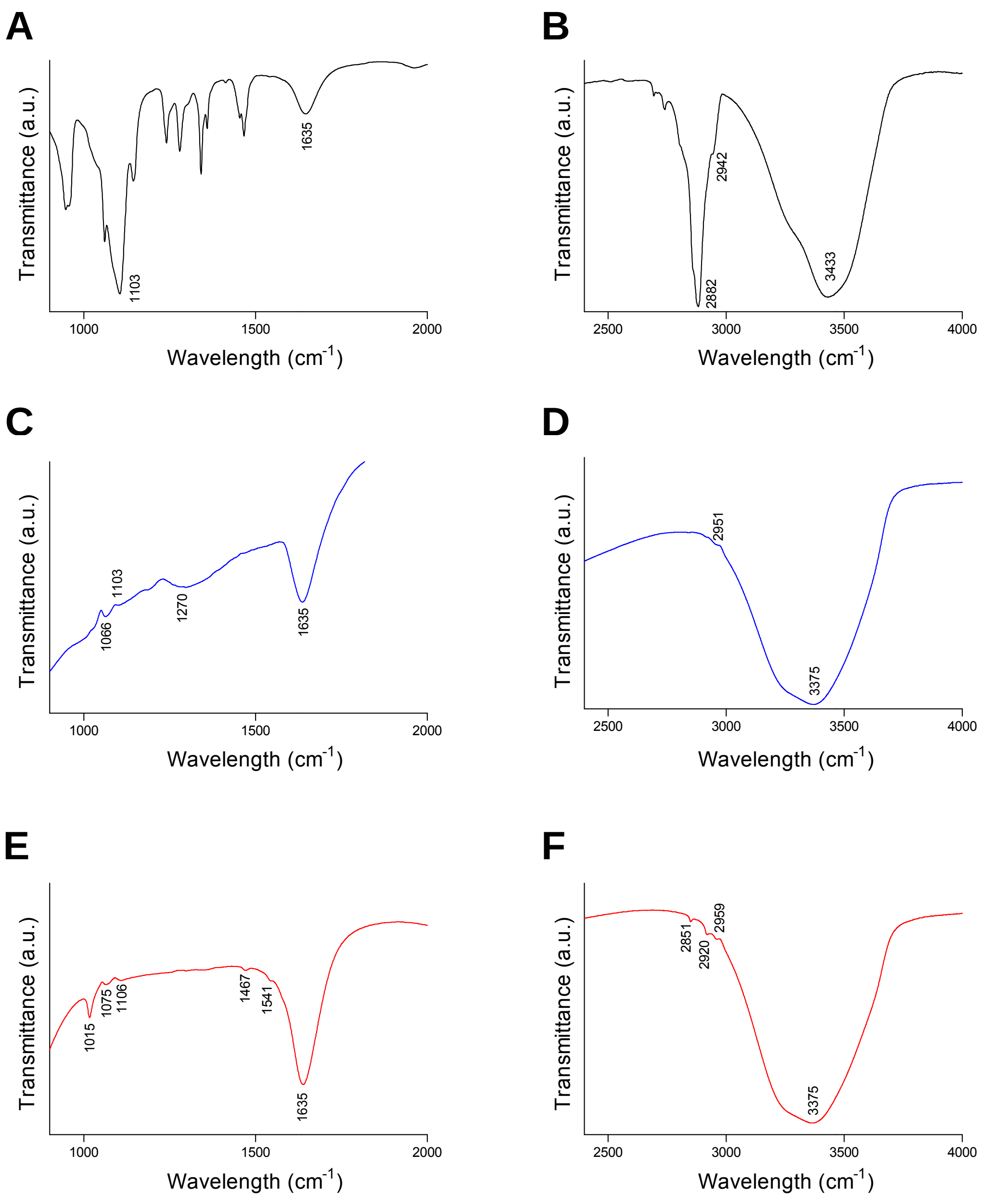

Figure S1. Fourier Transform Infrared (FTIR) spectra in two spectral regions, between 900-2000 cm^{-1} and 2400-4000 cm^{-1}, are shown for (a, b) methyl-terminated poly(ethylene) glycol (PEG); (c, d) oxidized SWCNTs; and (e, f) PEG functionalized ox-SWCNTs. The spectrum of pure PEG is characterized by the stretching vibration of the

C–H at 2882 cm^{-1}, the C=O stretching vibration at 1635 cm^{-1}, the O–H bending vibration at 1385 cm^{-1}, the deformation vibration of the C–H bonds at 1468 and 1342 cm^{-1}, the bending vibration of the O–H at 1280 and 1242 cm^{-1} and the C–O stretching vibration at 1149 cm^{-1}. FTIR spectra showed C-O stretching bands at 1066 and 1270 cm^{-1} for c-SWCNTs and at 1075 cm^{-1} for PEG functionalized c-SWCNTs (PEG-c-SWCNTs). The results also showed C-O-C bond stretching at 1103 cm^{-1} for c-SWCNTs and 1106 cm^{-1} for PEG-c-SWCNTs. Additionally, the PEG-c-SWCNTs showed peaks for interplanar stretching of aromatic rings at 1015 cm^{-1} and C-O-H bond stretching peak of carboxylic acid at 1467 cm^{-1}. Presence of the aforementioned bonds confirmed extensive carboxylation on the surface of the c-SWCNTs and PEG-c-SWCNTs. Attachment of PEG on the surface of the c-SWCNTs was done through the process of amidation representing covalent attachment (peptide bond) of PEG onto the surface of the c-SWCNTs. We observed the presence of Amide II bond peak in the PEG-SWCNTs FTIR spectrum at 1541 cm^{-1} representing the overlap of the N-H bending and C-N stretching vibration and confirming the covalent attachment of the PEG chain to the surface of the c-SWCNTs. Furthermore, the 1541 cm^{-1} peak was absent in both the PEG only and ox-SWCNTs spectra. Presence of the 5kDa PEG chains on the surface of the PEG-c-SWCNTs was further verified through the detection of the PEG characteristic methylene C-H stretch peaks present at 2882 and 2942 cm^{-1}, in PEG-SWCNTs spectrum at 2837 and 2914 cm^{-1} representing symmetric and asymmetric CH_2 stretching, respectively. CH_3 asymmetric stretching peaks were also observed in the ox-SWCNTs and PEG-SWCNTs spectra at 2951 and 2959 cm^{-1}, respectively representing the backbone structure of the SWCNTs. Since all the samples were suspended in distilled water, two characteristic peaks of H_2O, *i.e.,* H-O-H scissor at 1635 cm^{-1} and OH stretching at 3433

cm^{-1} (PEG) and 3375 cm^{-1} (c-SWCNTs and 5kDa PEG-c-SWCNTs) were found in all samples.

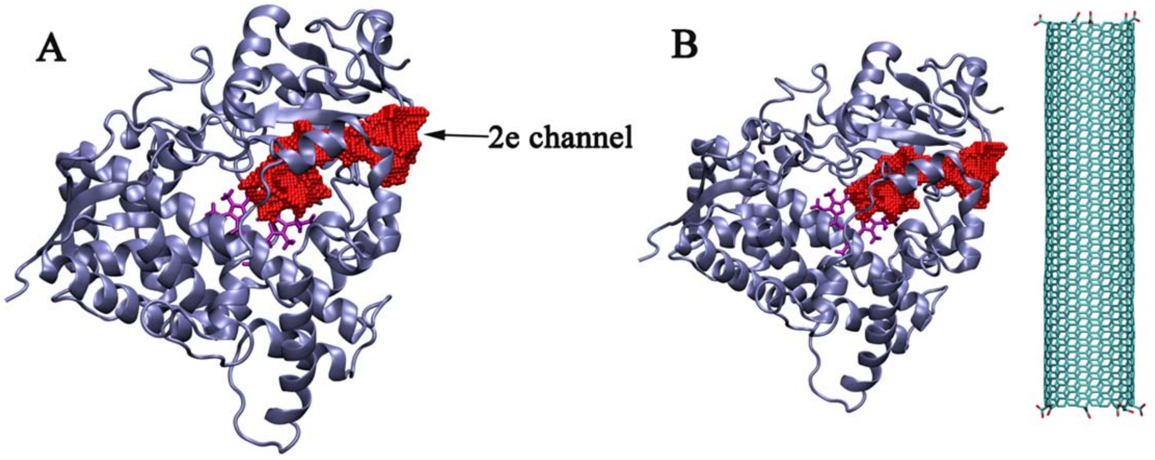

Figure S2. CYP3A4 (a) is shown with secondary structure colored in blue and the 2e channel is illustrated with red van der Waals (vdW) balls. (b) The initial configuration of Model 1 is shown.

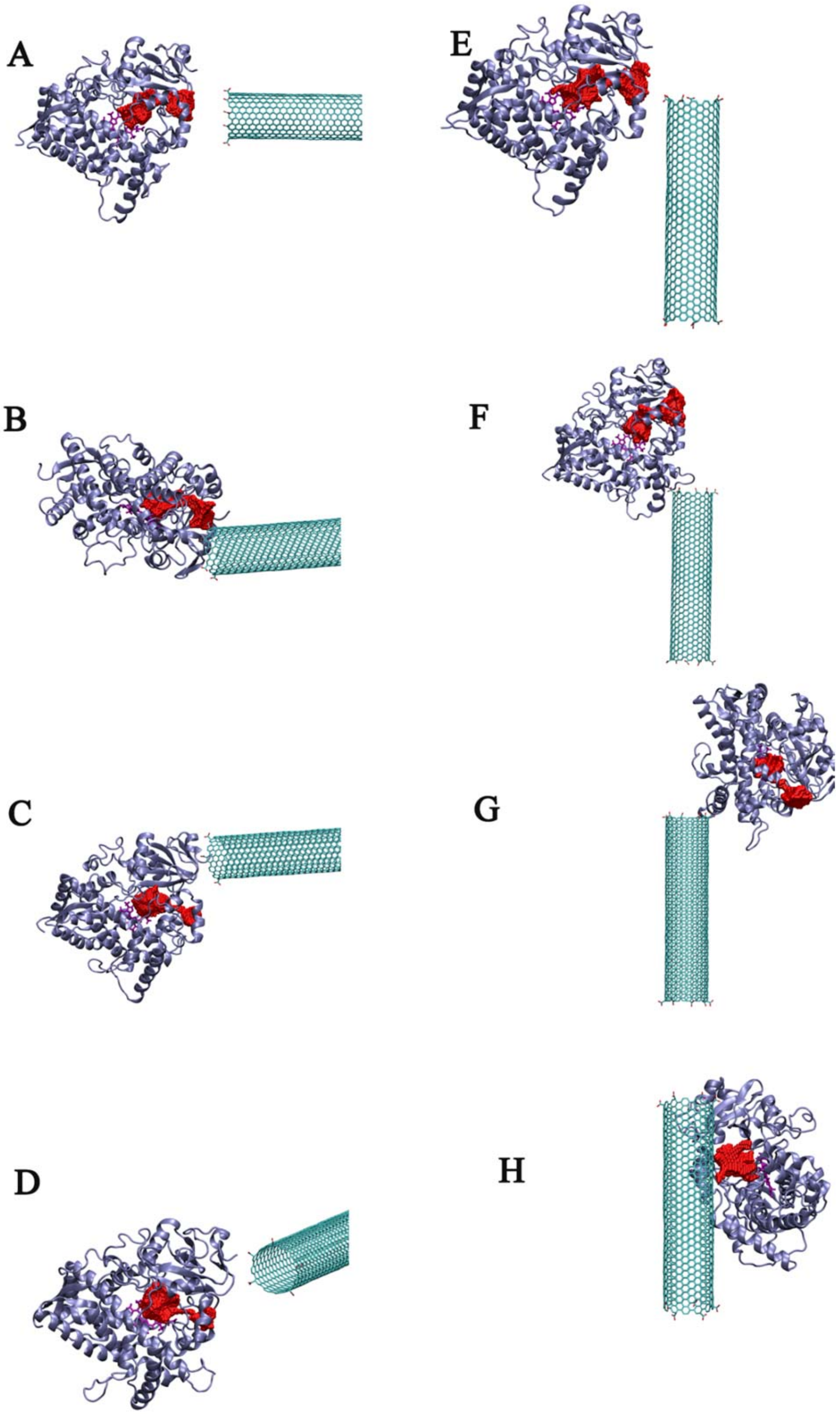
A
E
B
F
C
G
D
H

Figure S3. Two other molecular dynamics models (Model 2 and Model 3) with different initial configurations (each running for three independent trajectories). The initial configurations with c-SWCNT heading directly to the 2e channel (Model 2; a) and the c-SWCNT edge approaching the 2e channel from side (Model 3; e) are shown. The last frames of two configurations running for 100 ns are shown (b-d, f-h). Wherein, the active center is shown by purple sticks and the 2e channel is indicated with some red vdW balls.

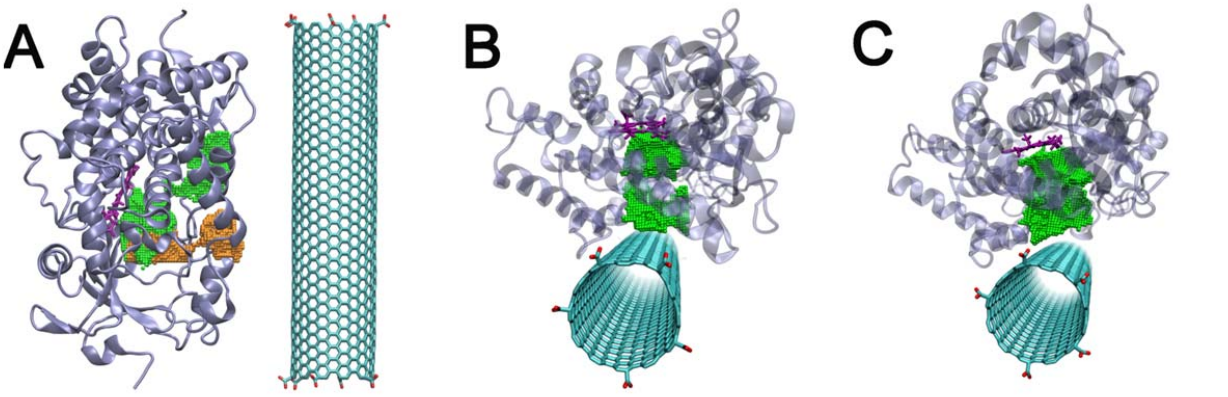

Figure S4. Model 4 with the c-SWCNT faced to the 3 (orange vdW balls) and S (green vdW balls) channels. The initial setup (a) and the two final snapshots (b and c) at t = 120 ns from two independent trajectories. The entrance for the 3 channel is shown with orange surface.

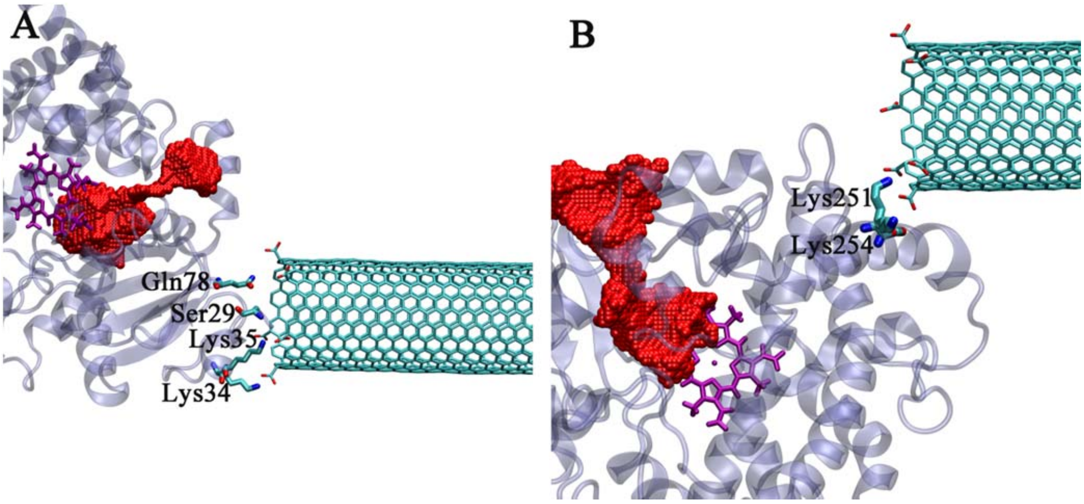

Figure S5. Two representative local snapshots of two trajectories chosen from Model 2 (a) and Model 3 (b). The key binding residues (hydrophilic and basic residues) in CYP3A4 are shown.

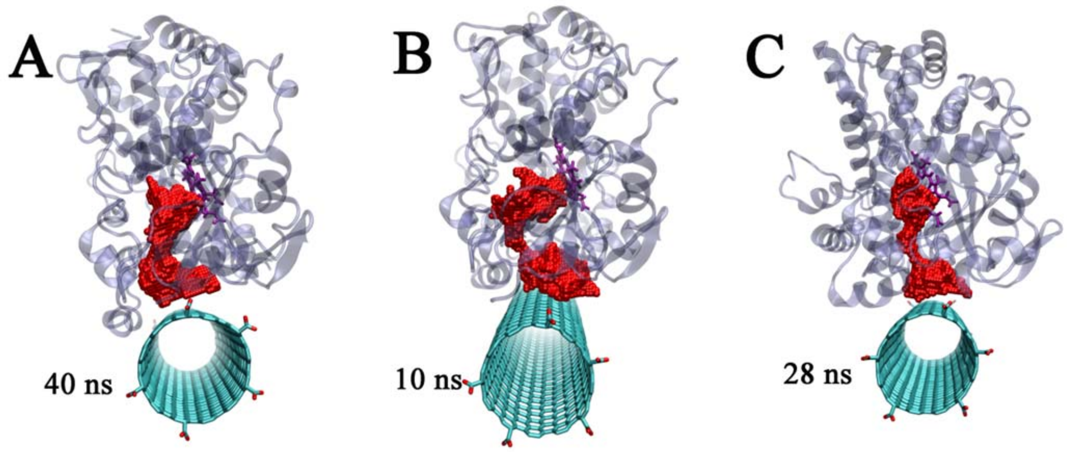

Figure S6. MD simulations of c-SWCNT and CYP3A4. (a-c) The timescale for the start of the 2e channel blocking by the c-SWCNT in all the three independent runs of Model 1. Once blocked, the 2e channel will keep the blocked state unchanged until the end of the simulation.

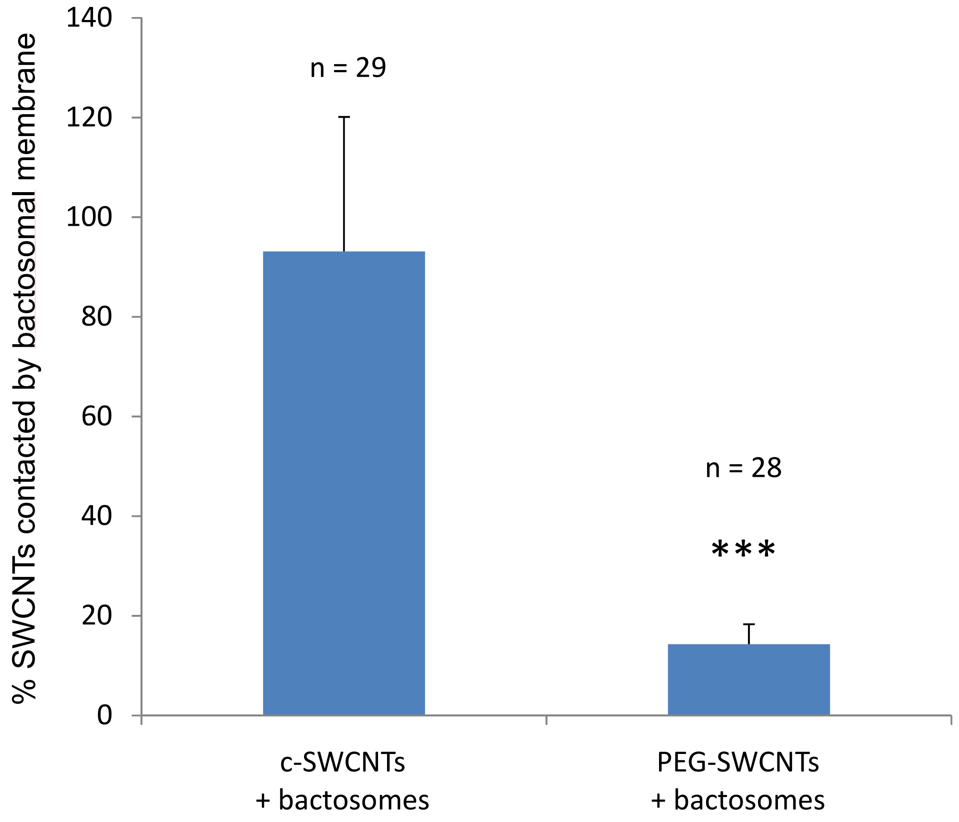

Figure S7. Effect of PEGylation on protein interactions. CYP3A4 interaction with the side-wall of the c-SWCNTs and PEG 5kDa-c-SWCNTs is presented as 'percentage of SWCNTs covered by bactosomal membrane'. The percentage was calculated using treated *versus* untreated samples (refer to AFM images depicted in Fig. 6). Statistical analysis to demonstrate differences in CYP3A4 binding to the side-walls of c-SWCNTs and PEG 5kDa-c-SWCNTs was performed using unpaired Students t-test (*** p value <0.001).
